# The impact of intraoperative “Nerve Monitoring” in a tertiary referral center for thyroid and parathyroid surgery

**DOI:** 10.3389/fsurg.2022.983966

**Published:** 2022-08-10

**Authors:** Pietro Princi, Gaetano Gallo, Serena Elisa Tempera, Antonio Umbriano, Marta Goglia, Federica Andreoli, Casimiro Nigro

**Affiliations:** ^1^ UOC Centro Multifunzionale di Chirurgia Endocrina, Ospedale Cristo Re, Roma, Italy; ^2^ Department of Surgical Sciences, Sapienza University of Roma, Roma, Italy; ^3^ Operative Unit of Surgery, Cerignola Hospital, Foggia, Italy; ^4^ Department of Surgery, Sant'Andrea Hospital, Sapienza University of Roma, Roma, Italy; ^5^ Department of General Surgery, University of Rome “Tor Vergata”, Roma, Italy

**Keywords:** thyroid surgery, thyroidectomy, recurrent laryngeal nerve, intraoperative neuromonitoring (IONM), cost analysis, parathyroid surgery

## Abstract

The most fearsome complication in thyroid surgery is the temporary or definitive recurrent laryngeal nerve (RLN) injury. The aim of our study was to evaluate the impact of intraoperative neuromonitoring (IONM) on postoperative outcomes after thyroid and parathyroid surgery. From October 2014 to February 2016, a total of 80 consecutive patients, with high risk of RLN injuries, underwent thyroid and parathyroid surgery. They were divided in two groups (IONM group and control group), depending on whether neuromonitoring was used or not. We used the Nerve Integrity Monitoring System (NIM)-Response 3.0® (Medtronic Xomed®). The operation time (*p* = 0.014). and the length of hospital stay (LOS) (*p* = 0.14) were shorter in the IONM group. Overall mean follow-up was 96.7 ± 14.3 months. The rate of transient RLN palsy was 2.6% in IONM group and 2.5% in the control group (*p* = not significant). Only one case of definitive RLN injury was reported in control group. No differences were reported between the two groups in terms of temporary or definitive RLN injury. Routine use of IOMN increases the surgery cost, but overall, it leads to long-term cost savings thanks to the reduction of both operating times (106.3 ± 38.7 vs 128.1 ± 39.3, p: 0.01) and LOS (3.2 ± 1.5 vs 3.7 ± 1.5 days, *p* = 0.14). Anatomical visualization of RLN remains the gold standard in thyroid and parathyroid surgery. Nevertheless, IONM is proved to be a valid help without the ambition to replace surgeon's experience.

## Introduction

Thyroidectomy is one of the most frequent surgical procedures in general surgery Units (1) with about 40.000 thyroidectomies performed in Italy every year (66.66/100.000 inhabitants) (2). Unilateral or bilateral temporary or definitive recurrent laryngeal nerve (RLN) injuries are among the most frightening complications during thyroidectomy, leading to voice and swallowing alterations (3). In particular, the incidence of temporary unilateral RLN injury ranged from 1% to 20% with a rate between 1% and 2% in high-volume centers (4, 5)

The risk of RLN injury depends on the type of both surgical procedures and pre-operative diagnosis as well as surgeon experience (6). Unfortunately, most of RLN injuries are not visible to the naked human eye: on average only 1/10 lesions can be recognized during surgery (3–6). Moreover, to underline the complexity of this type of surgery, it is necessary to consider some factors that can increase the risk of RLN injury such as redo-surgery, giant or intrathoracic goiter, cancer and previous radiotherapy (7–11), without forgetting that the injury can also occur accidentally due to clamping, stretching, electrocautery or ligature entrapment (5, 12).

There is agreement that the gold standard approach to RLN is the accurate visual identification of the nerve (7, 13). The need of an easier and safer identification and dissection of RLN, also considering legal implications of surgical procedure complications, led some authors to suggest the introduction of routine intraoperative neuromonitoring (IONM) of the RLN during thyroidectomy (14–16). However, the real usefulness of routinely IONM is still debated, especially considering the cost of the equipment (6, 17–22).

The aim of our study was to evaluate the impact of IONM on postoperative outcomes after thyroid and parathyroid surgery for both benign and malignant disorders.

## Materials and methods

This was a retrospective single-center study of collected data from patients undergoing thyroid and parathyroid surgery from October 2014 to February 2016 and is reported according to the Strengthening the Reporting of Observational Studies in Epidemiology (STROBE) statement for cohort studies (23).

During the study period, 205 thyroid procedures were performed. The last 40 patients who underwent surgery without IONM (control group) and the first 40 patients who underwent IONM (IONM group), after its introduction into our daily clinical practice, were prospectively selected and included in the study.

All personal, anamnestic, and clinical-laboratory data of the patients were recorded. Anamnesis included the whole past medical history and in particular the pre-operative examination and diagnosis (thyroid fine needle aspiration, hormonal status, pre-operative use of drugs and a complete work-up of the performance status and the presence of eventual comorbidities). Data strictly related to surgery such as ASA score, type and duration of surgery, intraoperative electromyographic data, definitive histological diagnosis, postoperative length of hospital stay, and surgical complications were collected (LOS).

### Inclusion criteria

Patients with an increased risk of RLN lesions (10), including pre-operative anatomical abnormalities (non-recurrent nerve), were included in the study. All patients included in the study underwent a preoperative laryngoscopy to assess any lesion or voice alteration.

### Pre-operative diagnosis

Based on pre-operative diagnosis, patients were divided as follows: goiter (multinodular, giant goiter, intrathoracic); recurrent goiter; toxic goiter/Plummer's adenoma/Basedow's disease; primary hyperparathyroidism; suspected papillary carcinoma; papillary thyroid carcinoma; thyroid lodge abscess.

### Types of surgery

The following different types of surgery were performed: thyroidectomy; completion thyroidectomy; thyroid lobectomy; parathyroidectomy, thyroidectomy, and parathyroidectomy; thyroidectomy and central neck lymphadenectomy (LCC); thyroidectomy with central neck lymphadenectomy and unilateral lateral neck lymphadenectomy (LLC).

All patients received general anaesthesia with endotracheal tube insertion as required by the procedure and the related recommendations (24).

### Device monitoring and instrument settings

Nerve Integrity Monitoring System (NIM)-Response 3.0® (Medtronic Xomed®, Jacksonville, Florida, USA; REF 8253002, SN 2NR3-2055) with intermittent and continuous monitoring system was used in all cases. High contrast, digital, colour screen, 1024H × 768W pixels, Touch panel 256H × 256W. Impedance: <5 Ohm. Impedance difference: <1 kOhm. Stimulation level: 0.5 mA–1.5 mA (average 1 mA). Initial post-identification stimulation level: 0.5 mA. Frequency: 30 Hz. Stimulus duration: 100 µs. Minimum event threshold: 100 µV. The skin electrodes were placed in the pre-sternal region. The disposable monopolar (Medtronic Xomed) or polyuse (Neurovision Medical) stimulator probe was placed as a dissection tool.

### Standardization of intraoperative monitoring techniques

IONM was performed as previously described (25).

### Interpretation of the curve

The amplitude of the electromyography (EMG) curve relating to RLN stimulation depends on the following variables

Contact of the nerve stimulating probe and the presence of loose tissue and fascia covering the nerve, as well as the degree of humidity represented by the presence of blood have to be considered among those factors that may modify the amplitude of the EMG curve relating to RLN stimulation. Indeed, there are many variables that may play a role in the representation of the EMG curve such as the position of the laryngeal electrodes (in the eventuality of a response anywhere stimulated, it is suggested to scuff and reposition the endotracheal tube, because it is probably positioned too caudally), temperature, and dehydration of the nerve. All these eventualities have to be considered and well-known by the operator for a correct interpretation of the EMG curve.

The electrophysiological reference parameters of the intensity of the EMG response were as follows:

- Initial parameters: with mean stimulation at 1 mA, normal mean response of 900 µV (range 500–1800 µV).
- Final parameters: with final stimulation at 1 mA, average normal response of 1200 µV (range 150–5400 µV). Final event threshold averaged 0.37 mA (range 0.15–0.48).
- Parameters highly suggestive of recurrent lesion: with mean stimulation at 1 mA, response <200 µV or percentage reduction >50% compared to pre-dissection baseline control.

### Postoperative assessment

Patients with postoperative dysphonia or with significant reduction in EMG signal intensity post-dissection were evaluated with an otolaryngological visit and fibrolaryngoscopy performed on the second postoperative day to assess any lesions or changes in vocal cords motility. Subsequent post-operative checks, in case of dysphonia, were performed one month after surgery with a new phoniatric check-up, and 4–6 months after the operation with a vocal examination and eventual EMG repetition. Transient injury was defined as an injury in which the motility of the vocal cords recovered within 12 months after surgery.

### Statistical analysis

The usefulness of the intraoperative neuromonitoring system was assessed within the two groups using the following statistical tools: initially, a univariate analysis was performed on all potential factors, including the use of intraoperative nerve monitoring, influencing the recurrent deficit using Chi-square and Student's *t* test. Continuous data were expressed as mean ± standard deviation. Multivariate logistic regression was then performed. A value of *p* < 0.05 was considered statistically significant.

## Results

Demographic, clinical, and operative characteristics of the included patients are reported in Tables 1–3.

**Table 1:** Demographic and clinical characteristics of the included patients.

	All patients	C-group	IONM-group	*p* value
Patients	80	40	40	–
Age (±SD^a^) (range) years	50.6 ± 13.7	49 ± 16.5	51.4 ± 11.4	NS
Male/Female	21 (26.2%)/59 (73.7%)	10 (25%)/30 (75%)	11 (27.5%)/29 (72.5%)	NS
Mean follow-up time (±SD^a^) (range) months	96.7 ± 14.3	83.06 ± 5.41	110.30 ± 1.83	NS

^a^ SD standard deviation.

NS, not significant.

**Table 2:** Clinical characteristics and presentation of the patients enrolled.

Preoperative Diagnosis	All patients	C-group	IONM-group
Multinodular goiter	36 (45%)	13 (32.5%)	23 (57.5%)
Recurrent goiter	4 (5%)	2 (5%)	2 (5%)
Toxic goiter	9 (11.2%)	3 (7.5%)	6 (15%)
Primary hyperparathyroidism	1 (1.2%)	1 (2.5%)	0
Suspicious thyroid cancer	17 (21.2%)	14 (35%)	3 (7.5%)
Thyroid cancer	12 (15%)	6 (15%)	6 (15%)
Thyroid lodge abscess	1 (1.2%)	1 (2.5%)	0

**Table 3:** Procedures performed.

Surgical procedures	All patients	C-group	IONM-group
Total thyroidectomy	58 (72.5%)	27 (67.5%)	31 (77.5%)
Completion thyroidectomy	4 (5%)	2 (5%)	2 (5%)
Thyroid lobectomy	2 (2.5%)	2 (5%)	0
Parathyroidectomy	1 (1.2%)	1 (2.5%)	0
Total thyroidectomy + Parathyroidectomy	1 (1.2%)	0	1 (2.5%)
Total thyroidectomy + Central Neck lymphadenectomy	11 (13.7%)	6 (15%)	5 (12.5%)
Total thyroidectomy + Central Neck lymphadenectomy + unilateral lateral neck lymphadenectomy	3 (3.7%)	2 (5%)	1 (2.5%)

The overall mean operative time was 117.2 ± 40.3 min. In particular, the duration of surgery was 106.3 ± 38.7 min and 128.1 ± 39.3 min (*p* = 0.014) in the IONM group and in the control group, respectively. The median thyroid weight was 36.85 grams (range 8.7–344.2 grams). IONM was performed in nerves at risk.

The length of hospital stay LOS was 3.5 ± 1.5 days. The hospitalization time was shorter in the IONM group (3.2 ± 1.5) than in the group without IONM (3.7 ± 1.5) even if this difference was not statistically significant *p* = 0.14.

Pathological characteristics and procedural results were reported in Tables 4, 5.

**Table 4:** Pathological characteristics and follow up of the included patients.

Histology subtypes	All patients	C-group	IONM-group
	80	40	40
Struma	37 (46.2%)	15 (37.5%)	22 (55%)
Follicular adenoma	7 (8.7%)	5 (12.5%)	2 (5%)
Parathyroid adenoma	1 (1.2%)	1 (2.5%)	0
Papillary thyroid cancer	27 (33.7%)	14 (35%)	13 (32.5%)
Papillary thyroid cancer + Lymph nodes metastases	5 (6.2%)	4 (10%)	1 (2.5%)
Papillary thyroid cancer + Parathyroid adenoma	1 (1.2%)	0	1 (2.5%)
Microfollicular carcinoma	1 (1.2%)	1 (2.5%)	0
Poor differentiated carcinoma	1 (1.2%)	0	1 (2.5%)

**Table 5:** Procedural details.

Mean operative time (±SD^a^) (range) min	117.2 ± 40.3	106.3 ± 38.7	128.1 ± 39.3	0.014
Mean hospital stay (±SD^a^) (range) days	3.5 ± 1.5	3.2 ± 1.5	3.7 ± 1.5	0.14
Transient laryngeal nerve palsy	4 (2.5%)*	2 (2.6%)*	2 (2.5%)*	0.97
Definitive laryngeal nerve palsy y/n	1 (0.6%)*	0*	1 (1.25%)*	0.32

^a^ SD, standard deviation.

*Considering only RLN at risk.

The mean follow-up was 96.7 ± 14.3 months.

There were 4 cases of transient RLN injury among all the considered nerves (4/157 = 2.5%). In the IONM group the rate of transient RLN injury was 2.6%, while in the group without the use of IONM it was 2.5% (*p* = 0.97).

The four cases were one patient with thyroid carcinoma and one with benign goiter in each group respectively.

In the control group there was only one case of definitive recurrent RLN injury out of the total number of nerves considered (1/157 = 0.6%) *p* = 0.32. In this specific case, the patient was found to have hypomobility of the right vocal cord at otolaryngologic post-operative control; despite this, there was a complete functional recovery of phonatory activity, also reported by the patient, thanks also to early logopedic therapy.

### General cost analysis

The cost analysis was based on the economic assessments of the specific period in which the study took place.

The hospitalization of a patient who underwent a traditional total thyroidectomy cost around 2.400 € considering 3 days of hospital stay. These costs comprehend drugs (about 100 €), operating room equipment (about 130 €), diagnostic tests (about 160 €), hospitalization/discharge (about 50 €), ward overheads (about 80 €), medical care (about 250/216 € without and with IONM), nursing care (about 380/328 € without and with IONM), operating room staff (about 450/373 € without and with IONM), and operating room use (about 800/663 € without and with IONM) (26). Moreover, the cost of the intermittent disposable NIM kit, of about 440 €, has to be considered.

The application of IONM technique led to an increase in expenses because of the use of disposable NIM kit. However, reduction in operative time and LOS (even if not statistically significant) led to a reduction in the overall health care expenditure in the long-term.

Lastly, according to the Diagnosis Related Group (DRG), used in Italy to evaluate the reimbursement that each hospital can receive from the National Health System (*Sistema Sanitario Nazionale* or SSN), the average DRG for total thyroidectomy is 3340 €. This value allows us to define how in both groups there was a positive economic balance independent of the use of the IONM.

## Discussion

Several international experts highlighted the advantages of a selective use of IONM in case of thyroidectomy for cancer with lymph node dissection, autoimmune pathology, giant goiters, or in redo-surgery (27–31). Nevertheless, its constant application in the clinical practice is still debated (19).

The present study highlights how the use of the IONM, regardless of the malignancy or benignity of the disease, represents an effective tool to obtain a safer approach and a related overall reduction of health costs. Despite the increased time of setup and increment in costs due to the technology, there is a clear long-term benefit given by the correct intraoperative identification and evaluation of nerves functionality by the routine use of IONM. The benefits are especially evaluated in patients at risk of RLN injury where the visual identification could be more difficult.

Our results were consistent with those described by several authors (9, 24, 25, 32–36).

Calò et al. (25) reported their experience in 2.034 consecutive patients comparing identification alone versus IONM. According to the authors, the use of the IONM did not statistically affect the risk of RLN injury (28 RLN injuries/993 patients in the control group; 23 RLN injuries/1.041 patients in the IONM group) but can be useful in those patients with risk factors.

Staubitz et al. (32) published the results of the EUROCRINE database evaluating the impact of IONM on postoperative vocal cord palsy in 4.598 first-time thyroidectomies performed in 82 hospitals for benign disease between May 2015 and January 2019. There were 50 vocal cord palsies (1.1%) with a lower risk in the IONM group [Odd Ratio (OR) 0.34]. Interestingly, high-volume hospitals had a low injury rate (OR 0.05).

Considering the results obtained in our series, despite the small sample size, we agree with a recent systematic review (35) including 10 articles and 4.460 nerves at risk in the group with visual identification alone and 6.155 nerves at risk in the IONM group. The authors pointed out that the injury rate is lower after IONM in patients undergoing thyroidectomy for malignancy (3.5% vs. 2.1%, *p* = 0.050) with a considerable improvement even in those patients who had to undergo a redo-surgery (7.6% vs. 4.5%, OR: 1.32, *p* = 0.021).

For what concerns cost-benefit analysis of IONM employment versus visual identification of the nerves, Rocke et al. (33) reported a detailed cost utility analysis. They registered a general more cost-effectiveness of the visualization alone with cost savings of $179.40 and $683.20 per patient, but they also reported that if a clinician can, with use of IONM, decrease the rate of RLN injury by 50.4% or more compared with visual identification, selective use of IONM in high-risk cases is most cost-effective and should be suggested.

A recent Cochrane review of Cirocchi et al. (34) regarding the use of IONM or visualization alone in terms of patients' safety, analyzed 4 RCTs including a large population study of 1.558 patients. The authors reported no significant difference between the visualization-only and the IONM techniques in terms of rate of nerve injury, and they recorded a comparable duration of surgery in both cases. Despite these results, the authors reported, among the limitations of the study, a lack of standardization in the collection of data of the various trials that may have influenced the results. As suggested by Cirocchi et al. a well-designed, executed, analyzed and reported RCT with a larger population and longer follow-up, employing the latest IONM technology and applying new surgical techniques is needed.

This study has some limitations. The main limitations are represented by both the retrospective nature of the study and the lack of the use of standardized questionnaires such the Voice Handicap Index-10 (VHI-10) or the Impairment Index-5 (VII-5) valuation questionnaires that could make the results objectified with the existing Literature (36). It was not possible to compare the weight in grams of the thyroid with the postoperative outcomes due to the great variability of the specimen and for the lack of a real definition of weight ranges. Furthermore, the sample size was small, and no statistical matching was performed. However, these limitations were offset by having the procedures performed in a referral center with the observational nature of the data reflecting the current clinical scenario of most of the hospitals distributed worldwide.

Furthermore, considering the incidence of legal malpractice with lawsuit related to the recurrent laryngeal palsy in near of 57% of all cases in a referral center, as reported by Dralle et al. (37), the use of nerve monitoring in selected cases should be advisable even if, as showed by Abadin et al. (38), there is not clear evidence that the “use or nonuse” played a role in malpractice ligation related to thyroid surgery.

## Conclusions

The IONM allows a prognostic evaluation of the post-operative vocal cords functioning, preventing the possibility of bilateral damage, and permit to know the exact point and the cause of the RLN injury. However, the visual identification of recurrent laryngeal nerve remains the gold standard in case of neck endocrine surgery even if the use of intra-operative nerve monitoring is advisable.

## Data Availability

The original contributions presented in the study are included in the article, further inquiries can be directed to the corresponding author.
